# Scaff's Leather and Rubber Boots

**Published:** 1890-04-26

**Authors:** 


### HYGIENE.

### SCAFF'S LEATHER AND RUBBER BOOTS.

The history of shoes, like that of many other articles of common use, is interesting. For shoes and boots as we now have them were not produced at once, but have arrived at their present progress to perfection by many transitions. Doubtless some protection to the feet has been used from the days probably of primæval man. Herodotus tells us the Greeks and Romans had both shoes and sandals, the former covering the whole foot, the latter fitting the sole and secured with thongs. From the sandal and cover for the foot of untanned hide, to the wooden shoe, the clog, the patten, and the shoe and boot of the present day represents a period of centuries. It would appear that innovation in the matter of shoes shared the fate of many other improvements, not being well received. For Shakespeare in Henry VI. has it, "Spare none but such as got with clouted shoon, for they are thrifty honest men." "Popes and byshops," however, seem to have been allowed or even expected to "keepe their feete ful cleane with shoes of gold and silver." But, notwithstanding all improvements in the art of shoe-making, a perfect shoe is still a desideratum; for what is required is undoubted security against outside wet and damp, combined with ventilation inside. As everyone knows, there is scarcely any more fertile source of cold than a damp shoe, and this dampness may not only arise from the leather being wet through, but also from the natural perspiration of the foot accumulating inside producing dampness and cold immediately the individual becomes inactive. There is also no more fertile cause of corns, bunions, and disfigured feet than a badly fitting shoe or boot. Sydney Smith said we should never find out in this world why shoemakers always made our shoes to hurt us. And, without altogether endorsing this, we certainly never put on a pair of new shoes or boots without trepidation, and assuredly never essay to walk far on that day. In former times the law of England took cognisance not only of the quality of the leather which the shoemaker wrought into his goods, but also of the number of stitches that he furnished. But we are above all that now, and *caveat emptor* is the order of the day. Perhaps indifferent leather and inferior workmanship may account for the immense number of boots and shoes which are now imported from the Continent. Some years back an outer sole of gutta percha was announced as the panacea against damp, but the plan did not succeed well, the gutta percha frequently coming off wholesale and wearing out rather quickly. We have lately noticed an advertisement of "Scaff's patent leather and rubber combination boots," Leeds. The peculiarity is this. The sole consists of three parts. Next to the "uppers" is a leather sole extending from the heel to the front; attached to this is a sole of vulcanized rubber, on the under surface of which is moulded a set of projecting discs. These pass through corresponding openings in a third, or outer, leather sole, and projecting beyond its surface, form a soft and secure footing. We have used these boots for some time, and can endorse the advantages with which they are credited. The sole is impervious to damp under all conditions. They are noiseless, and thus recommended for use by the police, which, as the inventors mention, would "turn the tables on the burgling tribe." They impart lightness and spring to the step. They are an effectual remedy to the wearing down of the outside of the boot heel. They do not "draw" the feet in summer. They wear longer than an ordinary leather sole. They can be made light for a lady and heavy for the sportsman. There is also another advantage attaching to the use of these boots which the inventors do not appear to have recognised. This is that the rubber renders the medical man or nurse able to walk on the bare boards of a hospital ward, or in any sick room, without making any noise at all, even although no special precautions are taken. There is no occasion to go on tip-toe, or to tread on one side, or to adopt a stealthy step, all contrivances to avoid noise which are often so irritating to a sick person.

